# Tail Docking and Ear Cropping Dogs: Public Awareness and Perceptions

**DOI:** 10.1371/journal.pone.0158131

**Published:** 2016-06-27

**Authors:** Katelyn E. Mills, Jesse Robbins, Marina A. G. von Keyserlingk

**Affiliations:** Animal Welfare Program, Faculty of Land and Food Systems, University of British Columbia, Vancouver, British Columbia, Canada; ETH Zurich, SWITZERLAND

## Abstract

Tail docking and ear cropping are two surgical procedures commonly performed on many dog breeds. These procedures are classified as medically unnecessary surgeries whose purpose is primarily cosmetic. Available attitude research surrounding these controversial practices has been limited to surveys of veterinarians and dog breeders familiar with both practices. The aim of this project was to: 1) assess public awareness of tail docking and ear cropping, 2) determine whether physical alteration of a dog affects how the dog, and 3) owner are perceived. In Experiment 1 awareness was measured using a combination of both explicit and implicit measures. We found that 42% of participants (n = 810) were unable to correctly explain the reason why tail docked and ear cropped dogs had short ears and tails. Similarly, an implicit measure of awareness (‘nature vs nurture task’), found that the majority of participants believed short tails and erect ears were a consequence of genetics rather than something the owner or breeder had done. The results obtained in Experiment 2 (n = 392) provide evidence that ear cropped and tail docked dogs are perceived differently than an identical dog in its ‘natural’ state. Modified dogs were perceived as being more aggressive, more dominant, less playful and less attractive than natural dogs. Experiment 3 (n = 410) is the first evidence that owners of modified dogs are perceived as being more aggressive, more narcissistic, less playful, less talkative and less warm compared to owners of natural dogs. Taken together, these results suggest that although a significant proportion of subjects appear unaware of the practices of tail docking and ear cropping in dogs, these procedures have significant impacts on how modified dogs and their owners are perceived by others.

## Introduction

‘Man’s best friend’, the dog, was domesticated by humans over 15,000 years ago [1] and since that time they have remained an important part of society in many cultures around the world. From pottery and other artefacts, a pattern of genetic selection can be observed beginning as late as 7500 years ago [2]. Since then intensive artificial selection has resulted in 152 officially recognized dog breeds [3]; which vary drastically both morphologically and behaviourally. It is estimated that 37–47% of households in the United States own a dog, equating to ~70–80 million owned dogs [4].

When looking for a potential dog, there are many factors to consider, one of which is personality [5]. Stephen and Ledger (2007) report that certain personality and behavioural traits are associated with a higher risk of relinquishment to shelters; some of which include chewing furniture, aggression, anxiety, fear and excessive barking [6]. Interestingly, there is little correlation between breed popularity and trait desirability. While personality is certainly an important factor, popular dog breeds do not display more desirable behaviour, live longer or have fewer health problems than their less popular counterparts [7]. Although personality may be an important reason for relinquishment, aesthetics seem to be a key factor in choosing a dog breed resulting in a dog breed industry that is largely appearance driven [8]. For instance, potential owners may prefer short-haired dogs to long haired or light coloured fur to dark. This preference for varied aesthetics in dog breeds is seemingly due to a shift in attitudes to less emphasis on utility of the animal to more of an aesthetic appeal [7]. This shift resulted in the formal recognition of ‘breed standards’, some of which were introduced in the 1800s [3]. Breed standards outline specific physical characteristics, such as color, height, and body conformation (e.g. see specific breed standards set out by the American Kennel Club (AKC) and the Canadian Kennel Clubs (CKC)). Along with this focused approach to genetic selection, some breed standards include specifics regarding the length of the tail or position of the ears; which in some breeds can only be achieved through surgical removal or alteration (e.g. tail docking and ear cropping).

In Roman times, dogs had their tails docked as a means to decrease the spread of rabies [9], while ear cropping was practiced to prevent ear damage during fighting and hunting [10]. Today the reasons given for these surgical alterations include prevention of tail injury [11], decreased ear infections, breed conformity and a breeder’s right to choose [12]. However, there is very little research assessing the validity of these assertions [10, 13]. Despite this lack of evidence, ear cropping and tail docking have become defining features of many dog breeds. Historically, approximately one third of recognized dog breeds have their tails docked [13], but to our knowledge there are no reliable estimates of the extent to which both of these procedures are performed today.

Increasing concern about animals and their welfare has led many to question the practices of ear cropping and tail docking. Many countries have introduced legislation that restricts, and in some cases even bans these practices. For example, the European Union Convention for the Protection of Pet Animals prohibits surgical operations including tail docking and ear cropping for non-curative purposes [14]. In Israel tail docking is banned for cosmetic purposes whereas Scotland implemented a complete ban as of 2003 [15]. There is great variation in the amount of restriction placed on these procedures around the world. In North America, organizations such as the American Veterinary Medical Association (AVMA) and the Canadian Veterinary Medical Association (CVMA) have official position statements opposing these practices for cosmetic purposes. As a result, some Canadian provinces (e.g. Newfoundland and Labrador, British Columbia) and U.S. states (e.g. Pennsylvania, Maryland) have even passed by-laws that prohibit tail docking and in some cases also ear cropping [10]. Additionally, notable veterinary textbooks no longer contain ear cropping procedural outlines [16].

Available social science research surrounding these practices has been limited to surveys of groups very familiar with both practices–namely veterinarians and dog breeders [17]. To our knowledge this is the first research to assess public awareness of two medically unnecessary procedures and how they impact perceptions of the modified dogs and their owners. The goals of our research were threefold: 1) to assess awareness of two medically unnecessary surgeries, tail docking and ear cropping in dogs, 2) to test whether modified and natural dogs are perceived differently and, 3) to test whether owners of modified and natural dogs are perceived differently.

This research was approved by the University of British Columbia’s Behavioural Research Ethics Board protocol # H15-00324. Informed written consent was obtained from all participants. Participants that did not consent were unable to access the online survey. Consent forms and recruitment documents outlined methods for maintaining confidentiality and a general description of the study methods and objectives. All experiments were created using Qualtrics (Provo, UT) online survey platform.

## Materials and Methods

### Recruitment

In all three experiments, participants were recruited using Amazon’s Mechanical Turk crowdsourcing service (MTurk). MTurk has been shown to result in more diverse samples than traditional means of recruitment [18,19]. Additionally, MTurk participants are more attentive than traditional subject pools [20].

## Experiment 1

The aim of Experiment 1 was to determine awareness of tail docking and ear cropping in well-known dog breeds. All participants completed an implicit task followed by an explicit task to assess their awareness.

### Participants

Participants (n = 810) were United States residents with a mean age of 44 years (median 30 years; range 18–68 years); 474 (59%) were men, 336 (41%) were women. Of the total participants, 318 (39%) were a primary caregiver of a dog.

### Procedure

**Fig 1:**
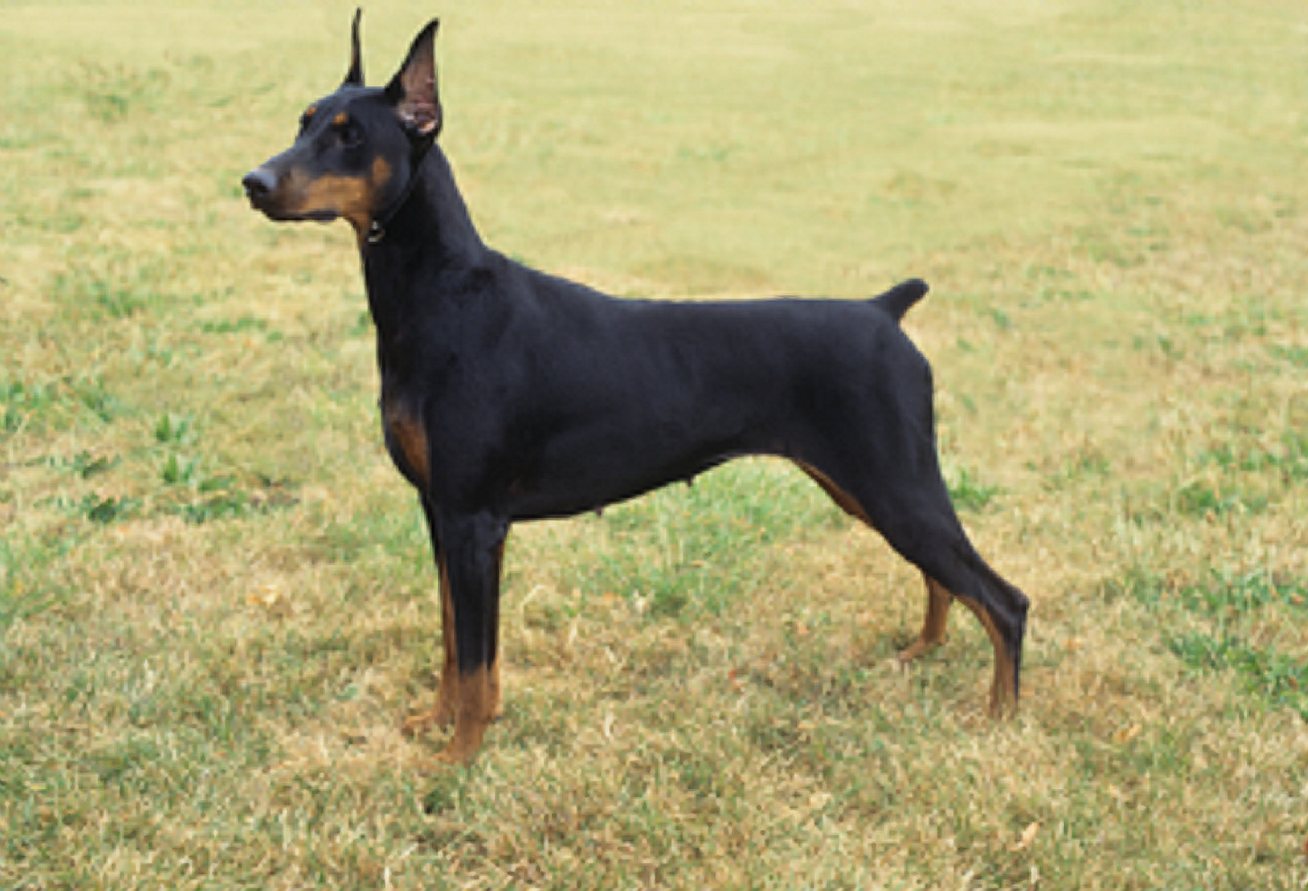
Example of full body image presented to participants in Experiment 1 to assess participants’ awareness of tail docking and ear cropping in dog breeds. Four dog breeds used as stimuli include Doberman Pinscher (pictured), Brussels Griffon, Boxer and Miniature Schnauzer. Photo by Mary Bloom with permission.

Four dog breeds that are commonly tail docked and ear cropped (Doberman Pinscher, Miniature Schnauzer, Brussels Griffon, Boxer) were chosen as stimuli for this experiment. These dogs were selected on the basis of their popularity according to AKC dog registration statistics [21]. Three of these breeds (Doberman, Boxer and Schnauzer) appear in the top 20 registered dog breeds. The fourth (Brussels Griffon) is not highly ranked (89^th^) but was selected in order to include a small ‘toy’ breed. Four full body images of individual dogs, representative of each breed were obtained from a professional photographer. An example of stimuli used can be seen in Fig 1.

#### Implicit measure

Participants were presented with a modified ‘nature vs nurture’ task used to assess lay beliefs about heredity [22]. Participants were told that they were participating in a study to test their understanding of heredity in dogs. They were then randomly assigned to evaluate one of the four dog breeds, accompanied by the statement, “This is [name] he/she is a 4 year old [breed]”. The name and gender of the individual dogs were the same for each breed. Next, they were presented with a list of 10 traits and asked to rate the extent to which they thought each trait was the result of either genetic or environmental factors using a 7 point Likert scale (0 = all genetics; 6 = all environment). Definitions of both genetics and environment were anchored at scale extremes (genetics: a trait the individual is born with–inherited from mother and/or father; environment: a trait the individual is not born with–result of being raised by owner or breeder). The traits participants rated were presented in random order and included: fur color, aggression towards other dogs, playfulness, fear of people, dominance, excessive barking, body size, number of teeth, and the two traits of interest- tail length, ear shape and size.

#### Explicit measure

After completing the nature-nurture task, participants were randomly assigned to receive two different images of the same breed of dog–one pictured in its natural state (long tail and ears) and one modified (cropped ears and docked tail). Participants were told “both of the above dogs are purebred [breed]. In fact, these two dogs are siblings, yet their ears and tails look very different” and then asked to select which one of the following three statements best explains this difference in physical appearance. Participants could select from the following three options:

Option A: Individual dogs of the same breed vary in appearance, meaning some will have tails and ears of different shapes and sizes.
Option B: Some dog breeds have part of their ears and tails surgically removed after they are born.
Option C: None of the above.
Options A and B were counterbalanced to minimize order effects. Option C always appeared last.

Finally, participants were asked to provide basic demographic information including age, gender, income and state of residence, if they were the primary caregiver of a dog and if they, or anyone they know, owned any of the dog breeds used as stimuli (Doberman Pinscher, Miniature Schnauzer, Boxer, Brussels Griffon).

## Experiment 2

Previous research has shown that physical appearance is utilized to make inferences about personality traits of others [23,24]. Experiment 2 sought to test if this is true in the case of dogs as well.

### Participants

Participants (n = 392) were United States residents with a mean age of 34 years (median 31 years; range 18–77 years); 241 (61%) were men, 151 (39%) were women. Of the total participants, 149 (38%) were a primary caregiver of a dog.

### Procedure

**Fig 2:**
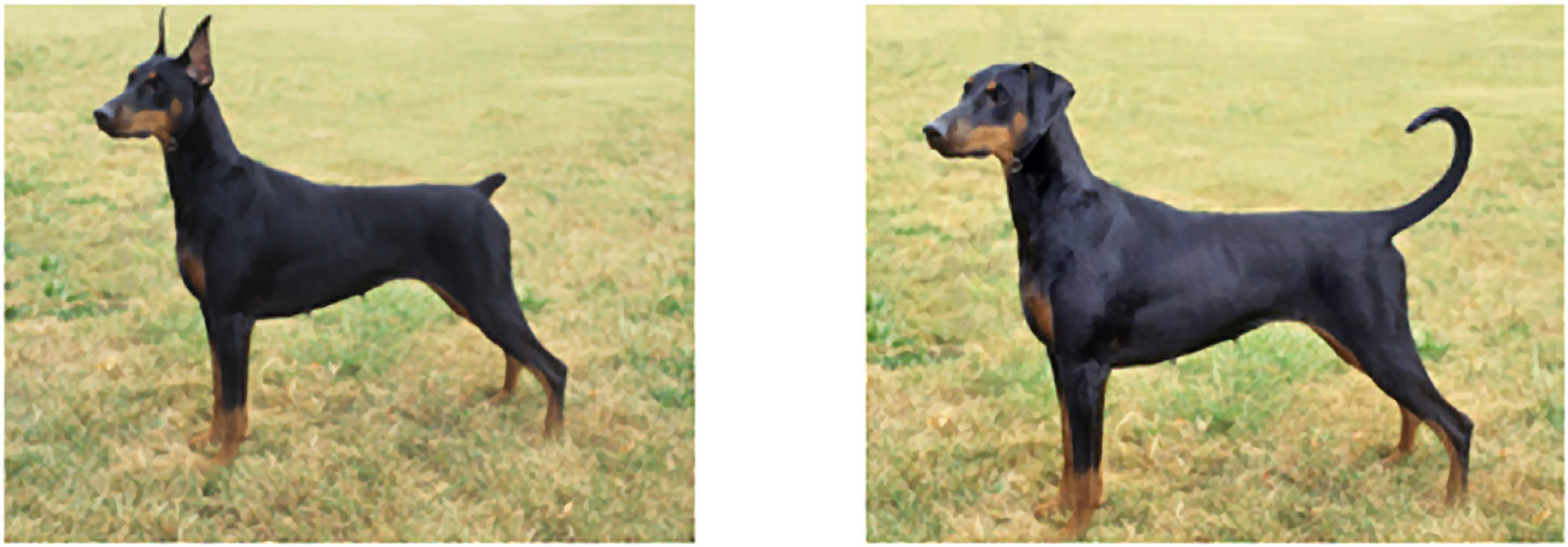
Example of natural and modified full body images presented to participants in Experiment 2 to assess participants’ perceptions of ear cropping and tail docking in dogs. Additional dog breeds presented to participants include Brussels Griffon, Boxer and Miniature Schnauzer. Photo by Mary Bloom with permission.

The same four images used in Experiment 1 were modified by a graphic artist to depict them as they would appear with their natural tail length and ear conformation (“natural”). Besides the addition of ears and a tail the images were identical (see Fig 2). In total there were 8 different images used as stimuli (4 dogs x 2 versions). Participants were randomly assigned to receive one version (natural or modified) of each breed, for a total of 4 images.

To conceal the aim of our study, participants were told that they would be participating in an experiment to see whether or not it was possible to accurately predict the personality of dogs using nothing more than a photograph. We told participants that we had created detailed personality profile for each of the dogs they were about to evaluate and that their evaluations would be compared to these profiles to see how accurate they were. To further enhance believability, we also mentioned actual research showing that evaluations of human personality traits based on very limited information can in fact be accurate [23,24]. Three instructional manipulation check questions (IMC) were included to ensure that participants had read and understood the instructions. IMC questions minimize satisficing, increasing the validity of study results [25].

Participants rated each of the four dogs on a 7-point Likert (*0 = not at all likely*, *6 = extremely likely*). Traits were selected from a previously published dog personality questionnaire [26].

Lastly, participants provided the same basic demographic information as described in Experiment 1.

## Experiment 3

Experiment 2 was designed to determine if participants make different judgments towards individual dogs, based solely on differences in tail length and ear conformation. To build upon this, Experiment 3 was designed to determine if owners of modified and natural dogs are perceived differently.

### Participants

Participants (n = 420) were United States residents with a mean age 34 years (median 32 years; range 18–79 years); 235 (56%) were men, 151 (44%) were women.

### Procedure

**Fig 3:**
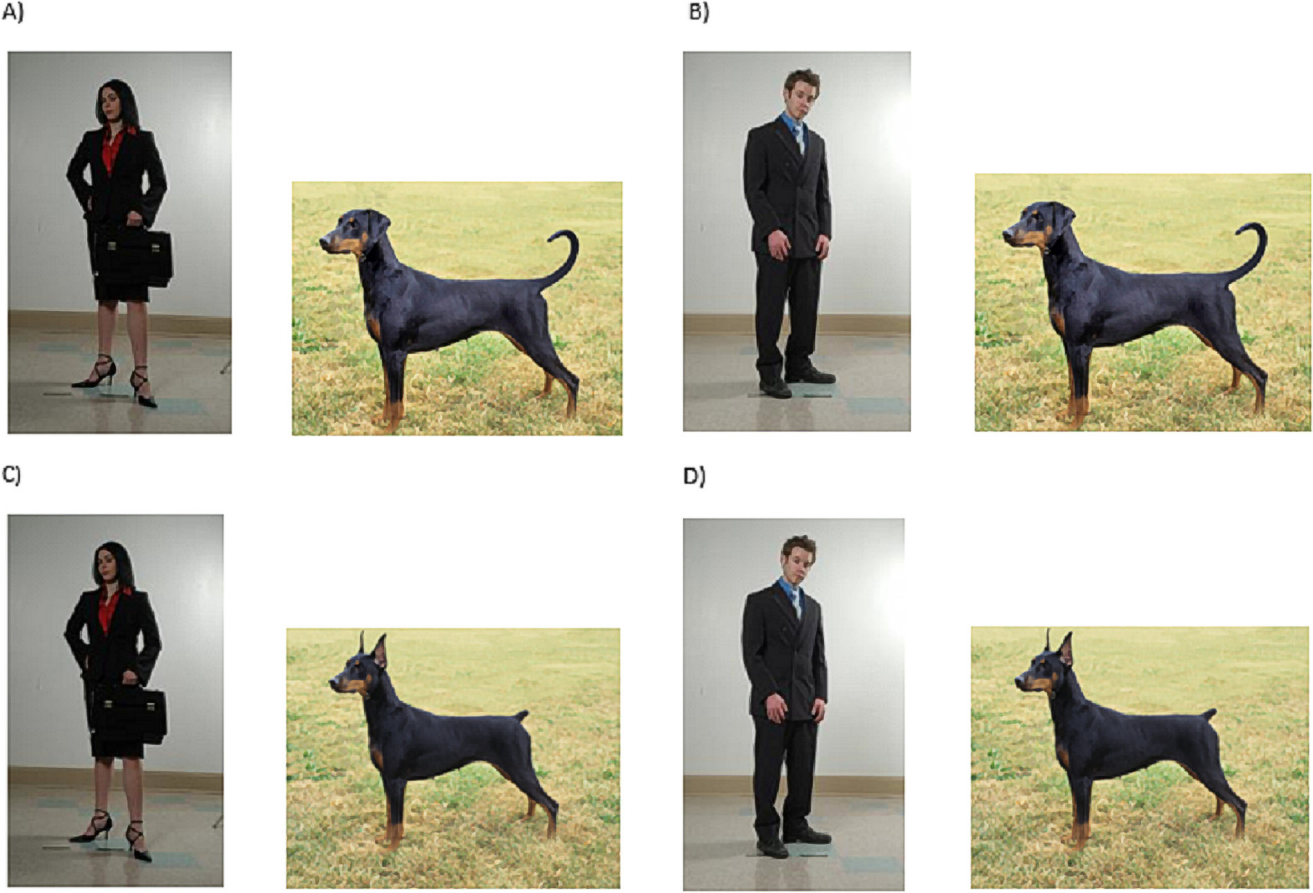
Stimuli presented to participants in Experiment 3 to assess participants’ perception of owners of modified or natural dogs. Images were randomized to ensure participants received 1) male dog owner with natural (B) or modified (D) Doberman Pinscher and 2) female dog owner with natural (A) or modified (C) Doberman Pinscher. Owner photos by Vidrio (https://www.flickr.com/photos/thatrileygirl/) is licensed under CC BY-SA 2.0 (https://creativecommons.org/licenses/by-sa/2.0/). Dog photo by Mary Bloom with permission.

Full body pictures of both a male and a female were used as owner stimuli. This owner stimuli was used in conjunction with a full body image of a Doberman Pinscher, both the original image (modified) and altered photo with natural ear and tail conformation (natural) (see Fig 3).

Participants were told that they would be participating in a study assessing whether an individual’s choice of pet communicates information about their personality. They were told they would be presented with a picture of a real person (Karen or Brian) and their pet (Pepper) and asked to answer a series of questions about the person. Participants were then randomly presented with a 2x2 design, gender of owner (male or female) and appearance of dog (natural or modified) were randomly varied. Our dependent variables included two broad dimensions of social perceptions, warmth and competence, in addition to a pet attachment scale and the single item narcissism scale (see Table 1). Additionally, we modified questions used in Experiment 2 to assess dog personality traits in order to determine if there were similarities in how dogs and owners were perceived.

**Table 1:** Personality traits assessed by participants for dog owners’ with natural or modified dogs on a 7 point Likert scale (0 = not at all; 6 = extremely).

Construct	Items	Adapted from
**Warmth**	How […..] is Karen/Brian?	Ashton- James et al.
	*[warm*, *honest*, *tolerant*, *sincere*, *trustworthy]*	(2014) [27]; Fiske et
		al. (2002) [28]
**Competence**	How […..] is Karen/Brian?	Cuddy et al. (2004)
	*[competent*, *capable*, *intelligent*, *confident*,	[29]; Fiske et al.
	*independent]*	(2002) [28]
**Pet**	Karen considers Pepper a member of the family.	Gonzalez Ramirez et
**Attachment**	Karen and Pepper have a very close relationship.	al. (2014) [30]
	Pepper is Karen’s best friend.	
**Narcissism**	Karen/Brian is a narcissist.	Konrath et al. (2014)
		[31]
**Experiment 2**	How [….] is Karen/Brian?	Jones (2009) [26]
**traits**	*[aggressive*, *easily frightened*, *dominant*, *playful*,	
	*talkative*, *compliant*, *attractive]*	

Finally, participants were asked to provide basic demographic information including age, gender, income and state of residence. Participants were also asked if they are the primary caregiver of a dog, as well as if they, or anyone they know, own a Doberman Pinscher.

## Results

### Experiment 1

#### Implicit measure

Average scores (Mean±SD) were calculated for both tail length and ear conformation. The average scores for all breeds were 1.87±1.53 and 2.39±2.02 for ear conformation and tail length, respectively, with slight variation within each individual breed (see Table 2). No other stimuli (e.g. familiarity with dog breeds, age and gender) were significant.

**Table 2:** Average (Mean±SD) responses obtained from participants (n = 810) when asked whether behavioural/physical dog traits were a result of genetics or the environment. Participants were asked to rate based on a 7 point Likert scale (0 = all genetics; 6 = all environment).

Trait	Miniature Schnauzer	Brussels Griffon	Boxer	Doberman Pinscher	All Breeds
Tail length	2.18±1.86	2.10±1.70	2.56±2.15	2.73±2.27	2.39±2.02
Ear conformation	1.61±1.96	1.72±1.27	2.10±1.73	2.03±1.78	1.87±1.53

#### Explicit measure

A majority 58% (n = 469) of participants answered correctly (i.e. “some dog breeds have part of their ears and tails surgically removed after they are born”) (see Table 3). Familiarity with dog breeds used as stimuli did not alter the results (P>0.05). Participants that self-reported as being a caretaker of a dog were more likely to answer the question correctly than those that were not dog owners, 68% vs 51% respectively. Of female participants 66% answered correctly, compared to 53% of male participants.

**Table 3:** The number (and percentage) of participants that responded when presented with two dogs of the same breed with ear and tail conformation variation and asked the reason for this. Multiple choice options included a) genetic variation, b) surgical removal after birth or c) none of the above.

Item	Genetic variation (%)	Surgical removal of ears and tail (%)	None of the above (%)
**All participants (n = 810)**	327 (40.4%)	469 (57.9%)	14 (1.7%)
Gender			
Female (n = 336)	112 (33.3%)	220 (65.5%)	4 (1.2%)
Male (n = 474)	215 (45.4%)	249 (52.5%)	10 (2.1%)
**Primary caregiver of a dog**			
Yes (n = 318)	95 (29.9%)	216 (67.9%)	7 (2.2%)
No (n = 492)	232 (47.2%)	253 (51.4%)	7 (1.4%)

### Experiment 2

Paired t-test analyses were performed to determine the statistical difference between modified and natural versions of each breed for each of the traits assessed. Data were analyzed with and without IMC questions and did not have a significant impact on the results. Individual breed knowledge, age and gender also did not have an impact on the results and thus the responses from all participants (n = 392) were pooled.

Modified dogs were seen as more aggressive both towards people and towards other dogs and more dominant than natural dogs (see Table 4). Natural dogs were also reported as more playful and more attractive than modified dogs. When the traits were analyzed for each breed individually, responses for the breed types investigated were similar with the exception of the Miniature Schnauzer (see Table 5).

**Table 4:** Mean responses obtained from an Internet based survey from participants (n = 392) when asked to assess traits for natural and modified dogs. Participants were asked to rate based on a 7 point Likert scale (0 = not at all; 6 = extremely).

Trait Assessed	Natural	Modified	P value
Aggressive towards people	3.2	3.4	0.0077
Aggressive towards dogs	3.1	3.4	0.0025
Fearful of people	2.9	2.9	
Fearful of dogs	2.4	2.5	
Dominant over dogs	3.4	3.6	0.0013
Playful	3.9	3.5	<0.0001
Barks excessively	3.7	3.6	
Obedient	4.0	4.1	
How attractive?	3.7	3.5	0.0222
How old?	4.2	4.4	

**Table 5:** Mean responses obtained from an Internet based survey from participants (n = 392) when asked to assess traits for natural and modified dogs for each individual breed. Participants were asked to rate based on a 7 point Likert scale (0 = not at all; 6 = extremely).

Trait Assessed	Boxer			Doberman Pinscher			Miniature Schnauzer			Brussels Griffon		
	Nat	Mod	P-value	Nat	Mod	P-value	Nat	Mod	P-value	Nat	Mod	P-value
Aggressive towards people	2.9	3.5	0.0004	3.4	3.9	0.0079	3.4	3.1		3.0	3.2	
Aggressive towards dogs	3.2	3.6	0.0055	3.3	3.8	0.0011	3.3	3.1		2.8	3.0	
Fearful of people	2.2	2.3		2.6	2.4		3.1	3.1		3.6	3.7	
Fearful of dogs	1.9	2.0		2.2	2.2		2.9	2.7		2.8	3.1	
Dominant over dogs	4.3	4.6	0.0480	4.0	4.6	<0.0001	3.2	3.0		1.9	2.4	0.0055
Playful	4.1	3.8	0.0294	3.9	3.3	0.0005	3.4	3.2		4.2	3.7	0.0001
Barks excessively	2.8	2.8		3.1	3.1		4.2	3.8	0.0098	4.7	4.6	
Obedient	4.7	4.6		4.8	4.9		3.7	3.7		2.9	3.0	
How attractive?	4.3	4.2		4.3	4.1		3.2	3.4		3.1	2.4	0.0001
How old?	4.0	4.0		3.8	3.8		5.4	5.5		3.8	4.3	

### Experiment 3

Paired t-test analysis was performed to determine the statistical difference between owners of modified and natural Doberman Pinschers, for each of the traits assessed. Significance was set at P<0.05. Individual breed knowledge, age and participant gender did not have an impact on the results (P>0.05) and thus the responses from all participants (n = 420) were pooled. Internal validity was high for all scales: 0.87 for warmth (5 items), 0.89 for competence (5 items) and 0.89 for pet attachment (3 items), which allowed us to collapse items.

Overall participants’ perceived owners of modified dogs as being more aggressive, more narcissistic, less playful, less talkative and less warm than owners of natural dogs (see Table 6). We also noted an effect of gender: the female owner of a modified dog was perceived by participants as being more aggressive, more dominant, more narcissistic and more competent than the female owner of a natural dog; whereas the male owner of a modified dog was perceived to be more narcissistic, less warm and less competent than the male owner of a natural dog (see Table 7).

**Table 6:** Mean responses obtained from an Internet based survey from participants (n = 420) when asked to assess traits of owners with either a natural or modified dog. Participants were asked to rate based on a 7 point Likert scale (0 = not at all; 6 = extremely).

Trait Assessed	Natural	Modified	P value
Aggressive	4.7	5.1	0.0042
Frightened	2.9	2.9	
Dominant	5.3	5.5	
Playful	3.8	3.5	0.0197
Talkative	4.5	4.2	0.0275
Compliant	4.1	4.0	
Attractive	4.7	4.6	
Narcissistic	4.2	4.8	<0.0001
WARMTH (5 measures)	4.4	4.2	<0.0001
COMPETENCE (5 measures)	5.6	5.6	
PET ATTACHMENT (3 measures)	5.5	5.4	

**Table 7:** Mean responses obtained from an Internet based survey from participants (n = 420) when asked to assess traits of owners (male or female) with either a natural or modified dog. Participants were asked to rate based on a 7 point Likert scale (0 = not at all; 6 = extremely).

Trait Assessed		Woman			Man	
	Natural	Modified	P value	Natural	Modified	P value
Aggressive	5.1	5.7	0.0013	4.3	4.5	
Frightened	2.6	2.4		4.2	3.3	
Dominant	5.8	6.1	0.0443	4.8	4.8	
Playful	3.3	3.0		4.3	3.9	
Talkative	4.5	4.2		4.6	4.3	
Compliant	4.1	3.8		4.2	4.3	
Attractive	5.2	5.2		4.2	4.0	
Narcissistic	4.4	4.9	0.0115	4.0	4.7	0.0023
WARMTH (5 measures)	4.3	4.1		4.5	4.2	<0.0001
COMPETENCE (5 measures)	5.9	6.1	0.0015	5.3	5.0	0.0012
PET ATTACHMENT (3 measures)	5.4	5.4		5.6	5.4	

### Discussion

#### Experiment 1

Our finding from Experiment 1 suggests that many people are simply unaware that the characteristic look of certain dogs is the result of surgical procedures. When participants were explicitly asked what explained different ear shape and tail length among dogs of the same breed slightly more, although a significant minority (42%) believed that tail length and ear conformation were a consequence of genetic variation. These results suggest that assessing public attitudes on contentious practices such as ear cropping and tail docking might be difficult given that a large minority of the individuals appear to be unaware that this practice takes place. The problem of non-attitudes, defined as a lack of strong or meaningful opinions on a matter, has been shown to result in attitude instability [32], which in turn can affect survey results [33]. This may also result in studies failing to truly reflect the publics’ attitude on a particular subject. Awareness studies can improve the ability to assess public attitudes as it attempts to account and arguably remove participants’ non-attitudes, resulting in more accurate results.

In the current study we observed that women appeared to be more explicitly aware that tail docking and ear cropping were not a result of genetic variation. This may be explained in part by women generally tending to be more involved in animal activism compared to men [34], which could subsequently mean women are more informed about these procedures. Not surprisingly, participants that were also primary caregivers of dogs were also more aware of these procedures compared to non-caregivers. Participants having no experience with dogs may be less aware (and arguably less interested in dogs per se) compared to those participants that did have experience with dogs. Additionally, given the natural variation in the length and type of tail across breeds, those participants with little experience with dogs may have considered the lack of the tail as being within the scope of the natural variation observed (e.g. some breeds such as the Australian Shepherd have naturally short tails).

#### Experiment 2

Experiment 2 showed that modified dogs are perceived as being more aggressive, more dominant, less playful and less attractive than dogs in their natural state. This is in line with other research that shows differences in coat color and ear conformation can change personality attributes given to dogs [35]. While the aforementioned study investigated how physical characteristics alter a dogs perceived personality, the current study aimed to determine if human induced physical characteristics in dogs negatively impact how humans perceive the dog. This distinction is important as human induced changes may negatively affect the perception and subsequently treatment of the individual animal.

Many of the traits that were perceived differently are ones that have been shown to predict both adoptability and relinquishment [36,37]. Reasons cited in support of ear cropping include supporting the appearance of fierceness or aggressiveness [38]; results of the current study support this perception. Anecdotally we see this supported in society in articles such as one entitled “11 riskiest dog breeds for homeowners and renters” [39], published by Forbes magazine in 2012 (Jersey City, NJ) where half of the dogs listed are traditionally tail docked and/or ear cropped breeds (Pit Bulls, Staffordshire terriers, Doberman Pinscher, Rottweiler, Great Dane, and Presa Canario). Interestingly the Forbes article also mentions that in the case of these 11 breeds, home insurance rates may increase or in some cases coverage may be denied.

Natural dogs were also perceived by the participants in our study to be more playful and attractive than modified dogs. Playfulness is an important trait when determining adoptability [37] and frequently a trait cited by owners as important when making the decision of which dog breed to buy [40]. This suggests that while the dog breeds used in this study are popular, they are not perceived to possess characteristics desired by owners (ie playfulness, attractiveness) and instead possess traits that typically result in relinquishment (ie aggression). The fact that the participants perceived the modified dogs to be less attractive than natural dogs is of interest and contradicts the reasons given by some that tail docking and ear cropping is needed; specifically, when argued by breeders that these procedures ensure the integrity and beauty of the dog breed [12]. Questions remain as to why these breeds are popular and if greater awareness that these dogs had undergone surgery to alter the tails and ears would negatively affect this popularity.

#### Experiment 3

To our knowledge this is the first study to provide evidence that physically altered pets affect the perceived personality and behavioural characteristics of their human owners. Aggression and playfulness were two of the traits used in Experiment 2 when assessing perception of dogs. In Experiment 2 the modified dogs were seen as less playful and more aggressive, which was similarly perceived in owners. This suggests that some behavioural traits perceived by participants in dogs are then reflected in their owners. This result supports the line of research that owners resemble their pets on some level [41]. In fact, studies have shown that participants can match dogs with their owners based on static images [42]. Therefore, increasing a dogs’ perceived aggressiveness by performing ear cropping and tail docking could in turn increase perceived aggressiveness in the owner which subsequently has the ability to affect human interactions. Furthermore, owners of modified dogs were perceived as less warm than owners of natural dogs. There is evidence to suggest that warmth is judged automatically, before competence, as whether a person appears to be friendly or trustworthy is more evolutionarily important than if they have the ability to act on these traits [43]. For example, viewing a person as more warm will more likely result in your desire to befriend the person or more simply approach the person than a person’s competence. In relation to the current study, this theory suggests that an owner of a modified dog appears more aggressive and less warm, resulting in an appearance that is less approachable than owners of a natural dog. Findings such as this that involve perception differences in personality are not exclusive to companion animals, in fact research has shown different houses and cars change perception of different gender roles and social status [44]. This is in line with a study by Wells and Perrine (2001) which found that a college professor with a dog or cat in their office was perceived as friendlier than if there was no animal [45].

Owners of a modified dog were also perceived as being more narcissistic than owners of a natural dog. Narcissism is described as “excessive self-admiration and feelings of superiority” [46] and narcissistic tendencies in humans having been linked to greater chances of social conflicts [47]. Taken together with the warmth and aggression results, it could be suggested that owners of modified dogs have a greater risk of social conflicts, human interaction complications and decreased approachability than owners of natural dogs.

## General Discussion and Conclusion

Dogs are a large and important part of society today. The current studies suggest that specific dogs with cropped ears and docked tails can be negatively perceived by the public and in turn this negative perception is reflected in their owners. In 2012, it was estimated that 70 million dogs are kept as pets in the United States alone [4]. This is a large number of individuals and while to our knowledge the number of ear cropped and tail docked dogs is unknown, they are among the most popular dog breeds [21]. With regards to animal welfare the question arises as to whether these negative perceptions affect adoptability and relinquishment of these dogs? As an example, approximately 3.9 million dogs enter shelters every year in the United States [4], with aggression cited as one of the top reasons for owners to relinquish their pets [6]. However, while relinquishment may not be a matter of ‘perceived’ personality traits, adoption is. When looking to adopt a new dog, potential owners are first drawn to appearance [48]. However, if the adoptable dog in question has cropped ears and a docked tail it can be assumed from the results of these studies that the potential owner will perceive this dog more negatively and this dog may be overlooked. Appearance based stereotypes are common in our society and with pets it is no different. Wright et al. (2007) determined that viewing one individual dog behaving badly or aggressively affected a person’s perception of the entire breed [49]. Many advocates for ear cropping and tail docking argue that the pain of the procedure has a very small impact on the individual. However, the results of this research show that by doing these procedures the perception and arguably treatment of these individuals is affected for their entire life.

Most interesting is that our findings indicate that by simply eliminating the procedures from these specific dog breeds we can shift the negative perception. Considering the lack of awareness of these procedures it appears that at least for those respondents that were unaware, the negative perception of short ears and short tail is unconsciously recognized. These three studies collectively provide evidence that human induced changes to a dogs’ appearance can dramatically affect how the dogs and their owners are perceived, which has the potential to negatively impact the dog as well as the owner. To our knowledge this is the first study that addresses the question of whether members of the public are aware that tail length and ear conformation of some breeds are a consequence of surgery and subsequently how these surgeries affect perception of dogs and their owners.

There are some limitations to the experiments presented. The results of these studies are based on a convenience sample of United States residents and therefore caution should be exercised when making any generalizations. Additionally, the dog breeds used in all three studies were limited in number and do not include all ear cropped and tail docked breeds. However, while this does mean that the results cannot be generalized to all dog breeds, we speculate that the concept could be applied to other breeds but strongly encourage future work to verify our findings across breeds. Similarly, one weakness with Experiment 3 is that it only used a single breed, the Doberman Pinscher. Clearly, further work must be done to see whether or not our findings can be generalized across other breeds that are ear cropped and tail docked to determine if the type of dog you own affects how others view you.

Taken together, the results of these three studies suggest that although a proportion of the participants that took our survey appear unaware of the practices of tail docking and ear cropping in dogs, these procedures appear to have profound impacts on how tail docked and ear cropped dogs and their owners are perceived.
